# Snapshots of Urban and Rural Food Environments: EPOCH-Based Mapping in a High-, Middle-, and Low-Income Country from a Non-Communicable Disease Perspective

**DOI:** 10.3390/nu12020484

**Published:** 2020-02-14

**Authors:** Mark Spires, Aravinda Berggreen-Clausen, Francis Xavier Kasujja, Peter Delobelle, Thandi Puoane, David Sanders, Meena Daivadanam

**Affiliations:** 1 School of Public Health, University of the Western Cape, Bellville 7535, South Africa; mark.spires@city.ac.uk (M.S.); pdelobelle@uwc.ac.za (P.D.); tpuoane@uwc.ac.za (T.P.); 2 Centre for Food Policy, City, University of London, London EC1R 1UW, UK; 3 Department of Food Studies, Nutrition and Dietetics, Uppsala University, 75122 Uppsala, Sweden; meena.daivadanam@ikv.uu.se; 4 Department of Epidemiology and Biostatistics, School of Public Health, Makerere University, Kampala, Uganda; fxkasujja@musph.ac.ug; 5 Chronic Disease Initiative for Africa, University of Cape Town, Cape Town 7925, South Africa; 6 Department of Public Health, Vrije Universiteit Brussel, 1090 Brussels, Belgium; 7 Department of Global Public Health, Karolinska Institutet, 17177 Stockholm, Sweden; 8 International Maternal and Child Health, Department of Women’s and Children’s Health, Uppsala University, 75237 Uppsala, Sweden

**Keywords:** food environment, low-, middle- and high-income countries, food retail outlets, food promotion

## Abstract

A changing food environment is implicated as a primary contributor to the increasing levels of non-communicable diseases (NCDs). This study aimed to generate snapshots of selected external food environments to inform intervention strategies for NCD prevention in three countries: Uganda (low income), South Africa (middle income) and Sweden (high income), with one matched pair of urban–rural sites per country. Fifty formal and informal food retail outlets were assessed, and descriptive and comparative statistical analyses were performed. We found that formal food retail outlets in these countries had both positive and negative traits, as they were the main source of basic food items but also made unhealthy food items readily available. The Ugandan setting had predominantly informal outlets, while the Swedish setting had primarily formal outlets and South Africa had both, which fits broadly into the traditional (Uganda), mixed (South Africa) and modern (Sweden) conceptualized food systems. The promotion of unhealthy food products was high in all settings. Uganda had the highest in-community advertising, followed by South Africa and Sweden with the lowest, perhaps related to differences in regulation and implementation. The findings speak to the need to address contextual differences in NCD-related health interventions by incorporating strategies that address the food environment, and for a critical look at regulations that tackle key environment-related factors of food on a larger scale.

## 1. Introduction

Mapping and research of food environments in general has to date primarily been carried out in high-income country (HIC) settings in response to the high prevalence of obesity and associated diet-related non-communicable diseases (NCDs) like type 2 diabetes [1,2,3]. Food environment evaluations are imperative in socioeconomically disadvantaged or otherwise vulnerable areas in HICs to promote equitable health outcomes, yet these diet-related diseases are also becoming increasingly prevalent in low- and middle-income countries (LMICs) [4,5]. There is rapidly emerging research on food environments in middle-income countries and a rather negligible amount on low-income countries [6]; hence an urgent need to develop and accelerate this research to address the cause and spread of these diseases in these parts of the world [3,7].

Food choices are affected by many factors in both the external food environment—presence of outlets and food availability within these, and factors related to the in-store environment, including quality, price, placement, and promotion [8,9]. There is more consistent evidence on the association between observed environmental factors and weight status, as compared to the direct link between dietary intake and environmental factors, which has shown varying results [10,11]. The availability of healthy food has been associated with a healthier diet, in particular when considering perceived availability [11,12,13]. Availability, accessibility and affordability are important determinants of consumer’s purchasing behaviours and store choice [9] and context influences shopping decisions through various triggers, with many decisions taking place at point of purchase [14]. In order to understand these interactions, it is necessary to study food environments within their context. Consequently, a LMIC-specific food environment conceptual framework developed by the Food Environment Working Group at the Agriculture, Nutrition and Health Academy (ANH-FEWG) [3] outlines separate, yet inter-related domains relevant to documenting and understanding food environments in these settings. The domains include: (1) external food environment, including all exogenous dimensions such as food availability, prices, vendor and product properties, and marketing and regulation; and (2) personal food environment, including all endogenous dimensions such as accessibility, affordability, convenience and desirability at the individual level. These two domains, and the dimensions therein, directly relate to and influence each other with regards to food acquisition and consumption, and ultimately health and nutrition outcomes [3]. The focus of this study was the creation of snapshots of external urban and rural food environments in selected sites in three low-, middle- and high-income countries—Uganda (UG), South Africa (SA) and Sweden (SW). The specific aim was to describe and compare selected aspects of these external food environments in order to inform the development of food-related intervention strategies for a larger implementation trial.

## 2. Materials and Methods

This study was part of the formative phase of a larger implementation research project in three settings, Uganda, South Africa and Sweden titled ‘A people centred approach to self-management and reciprocal learning for the prevention and management of type 2 diabetes’ (SMART2D) [15]. SMART2D strongly focuses on contextualization as a key factor in the development and implementation of self-management support interventions, recognizing the importance of the environment in which participants function on a daily basis as a key to intervention impact and sustainability [15,16,17]. In part through this present study, SMART2D seeks to understand the relationship between local food environments and food acquisition behaviours, and how this plays a role in managing type 2 diabetes (T2DM) in socio-economically disadvantaged or under-resourced areas. This study assessing the food environments was carried out as part of the context evaluations and used an NCD perspective, unhealthy diet being a common risk factor.

### 2.1. Study Design

This cross-sectional study was conducted to carry out observations of and collect data on the built environment related to food in selected sites within the three countries.

### 2.2. Sample

#### 2.2.1. Site Selection

The sampling process was carried out in two steps. In step one, the primary site was purposively selected from within the SMART2D trial intervention areas in each of the three countries, i.e., a rural area in Uganda, an urban township in South Africa and a socioeconomically disadvantaged suburb in Sweden. Primary sites were selected within communities identified by local researchers and stakeholders and defined as discreet geographical units with a central retail area. In step two, a comparable urban or rural counterpart matched to the primary site in terms of socio-demographic characteristics was selected, resulting in one urban and one rural setting per country. Step two was also carried out in consultation with local researchers, local demographic data sources and, in some cases, local government officials. In turn, this allowed for a comparable urban–rural sampling unit in the three countries and served to identify and highlight differences between the countries and the urban and rural areas where relevant.

#### 2.2.2. Description of Study Sites

The primary site in Uganda was a 1.3 km^2^ area of Mayuge, a rural town council in eastern Uganda, with a population of 17,392 and population density of 437 per km^2^ (2017) [18]. Approximately 63% of households in the district are involved in fishing, which is the main source of income. The district which includes the primary site had a population of 473,239—of which, approximately two-thirds of adults are illiterate. Approximately 6% of the households in the selected site have less than 2 meals a day [18]. The matched urban site is part of Kampala’s central business district and observations covered an area spanning approximately 0.4 km^2^. Kampala district as a whole had a population of 1,507,080 and a population density of 8562 people per km^2^ [19]. The district has lower illiteracy levels among adults (5.6%) and the proportion of households living on less than one meal a day in Kampala Central is approximately 12%. Most households depend on informal employment for their livelihood. Using diabetes as a proxy for NCDs, Uganda has an overall low prevalence (1.6%); that hides a range of prevalence estimates, one as high as 16.1% in a rural area and an overall pre-diabetes prevalence of 16% [20].

In South Africa, a 2 km^2^ area of Khayelitsha, a peri-urban township in Western Cape, South Africa, in the city of Cape Town was selected as the primary site (urban). In 2011, there were almost 400,000 residents in Khayelitsha, with a population density of 10,265 per km^2^, and 98.6% of them were Black African [21]. Just under 20% of residents were unemployed, with 30.8% of those over 20 having a high school diploma, and only 4.9% receiving any form of higher education [21]. For the matching rural site, a 7.5 km^2^ area that included Mount Frere and surrounding residential areas was selected. Mount Frere had 5252 residents, with a population density of 700 people per km^2^, 96.1% of them Black African [21]. Just under 11% of those residing in Mount Frere were unemployed, 38.2% of those over 20 had a high school diploma and 26% had some form of higher education [21]. South Africa has a high average age adjusted diabetes prevalence of 12.8%, with a high in-country variation, ranging from 3.8 to 14.7% [22].

In Sweden, the primary site (urban) covered an area of 2 km^2^ and included a ‘Million Homes Programme’ area in the Stockholm municipality [23]. The majority are immigrants living in ‘superdiverse’ communities, in social housing, with low incomes [24]. The area has a population of 18807, with a population density of 9403 people per km^2^—of which, 60.5% were born outside Sweden (2015); and an unemployment rate of 8.2% and 28% with higher education [25]. The matching rural site was the central town of Ljusnarsberg municipality covering 576 km^2^—of which, the town area included 6.7 km^2^ and 2 km^2^ of which was mapped; the total population of the municipality was 5006—of which, 3065 lived in the town with a population density of 457 per km^2^ [26]. In the municipality, 21% were born outside Sweden, 10% were unemployed and 11% had a higher education [27]. Sweden has a diabetes prevalence of 7.2% overall, lower than the European average of 8.9% [22], though immigrant populations have a significantly higher prevalence, 11.6% in one study among Iraqi immigrants [28].

### 2.3. Data Collection and Analysis

#### 2.3.1. Data Collection Tool

A semi-structured checklist and a systematic pre-defined process was used to carry out observations of the external food environment, capturing the characteristics that would be most relevant from the perspective of NCD interventions. Observations of local external food environments were carried out using a modified version of the Environmental Profile Of a Community’s Health (EPOCH) tool and methodology [29]. EPOCH seeks to capture, in a concise but generalizable form, environmental factors that may be associated with chronic disease risk factors, specifically heart disease [29].

The original EPOCH is a two-part tool consisting of a direct observation of different aspects of the built environment (infrastructure and services; commercial and shopping areas; grocery stores; tobacco retailers; restaurants; and pharmacy services) and a survey tool on community awareness, attitudes and social norms—of which, the first part was used for this study. EPOCH was adapted with permission for the purposes of this study, to make it more relevant for documenting food environments from the perspective of NCD prevention interventions (including T2DM) and food related activities, specifically focusing on self-management of T2DM. In the modified version, more food retail outlets were included and there was higher focus on fruits and vegetables, confectionaries and sweetened drinks, as well as the availability of healthier versions of a few daily items like bread, breakfast cereal, milk and yoghurt. To address our specific research objectives, the tool was adapted to include the following sections:

‘Community-wide tally of food retail outlets’—a new section developed to collect data on total food retail outlet tally and distribution in the selected sites.

‘Community observation walk’—a walk in a commercial or central shopping district designed to systematically observe and record food environment-related factors within a 1-km stretch considered by community members to be the main commercial district for services and facilities.

‘Food retail outlet assessment’ (‘assessment of a grocery store’ in the original tool)—in which the presence, price, and quality of fruits and vegetables, and certain packaged food products were noted, which was modified to include different types of outlets. The original tool was tested for reliability in five countries as part of the PURE study; inter-rater reliability was found to be good overall (24/38 excellent) [29]. In our modified tool, face validity was assessed based on discussions during the modification and piloting of the tool. The original tool was built on, to add relevant details pertinent to NCDs. This resulted in the addition of more outlets, food items and information related to specific food items. Content validity of the tool was assessed by (subject matter) experts working on the food environment and individual food consumption behaviour in the SMART2D consortium. The modified tool was sent to the experts in six partner institutions (from five countries) for them to judge whether sufficient aspects of the food environment were represented in the tool. Additionally, during the analysis conducted using the lens of the ANH framework, the tool was deemed to collect data related to each of the domains, further confirming the content validity of the tool.

#### 2.3.2. Data Collection Process

Data collection in each country was carried out by a team comprising one of the first authors (M.S.) and local investigators who had a background in health-related research and were familiar with the communities (F.X.K. and G.N. in Uganda and A.B.C. in Sweden). In-country orientation workshops of 1–1.5 days duration were conducted by M.S. to train local investigators and pilot the tools prior to data collection in the three settings. A standard training manual outlining the process and standard operating procedures (SOPs) for addressing specific issues that might arise was used during the training and data collection process. The adapted EPOCH tool was initially piloted in Cape Town, South Africa and Uppsala, Sweden before final amendments were made. Additionally, as part of the in-country orientation workshops, the tools were piloted in each setting with minor modifications being considered.

A process of ground-truthing, i.e., physical validation of observations was used to carry out two external food environment assessments per country, one each in an urban and rural area. Each assessment included one ‘Community Food Retail Outlet Tally’, one ‘Community Observation Walk’ and two ‘Food Retail Outlet Assessments’ per retail outlet typology (supermarket, independent grocer, convenience store, informal vendor, mobile vendor, and market). We included only those outlets that were present in the selected study sites, which implied that in some cases we were unable to carry out the requisite two assessments. Presence of food retail outlets for the ‘Community-wide tally of food retail outlets’ was mapped through a street-by-street walk in the selected sites to note all food retail outlets on the map. Based on this, a one-kilometer route was planned to include the busiest shopping area, and to include the maximum number of food retail outlets. Data were collected in the morning around the time shops open, using a paper version of the tools and a route map. The documentation began at the start point of the planned route, with the ‘Community Observation Walk’. When a retail outlet to be included in the ‘Food Retail Assessment’ was identified along the route, the data collectors approached the person in charge and asked for (oral) agreement to carry out the in-shop assessment then and there. When the ‘Food Retail Outlet Assessment’ of that outlet was complete, the ‘Community Observation Walk’ continued along the planned route, with further ‘Food Retail Outlet Assessments’ being carried out along the way. If the outlet was not available along the planned route, the nearest outlet to the walking route within the selected site was identified and observed. The observations included the presence of food product advertising, in-community and in-store; with health promotion advertisements included in the in-community observations. In case the assessments were not completed in one day, data collection was continued the next day and in the case of missing data, the concerned retail outlets were revisited.

For ‘Food Retail Outlet Assessments’, all fruits and vegetables available in the retail outlet were noted, as well as the general quality in terms of damaged produce. Per kilogram pricing information was recorded for the cheapest, not-on-sale item. In addition to fruits and vegetables, information was also gathered on pre-determined packaged food items. The packaged product with the lowest price per package was chosen for observation. In the case of two items of the same price, the healthiest option (in terms of sugar, fiber and fat content) was selected for grains (breakfast cereal and bread) and dairy (milk and yoghurt) and the added sugar version was selected for beverages.

#### 2.3.3. Data Analysis

All data were collected using paper instruments in the field with double data entry into REDCap (Research Electronic Data Capture) [30,31]. All study data were managed using REDCap electronic data capture tools hosted at Karolinska Institutet. Data analysis was carried out using IBM SPSS Statistics 24 (registered to Uppsala University). Descriptive statistical analyses were conducted on the food environment-related characteristics and relevant point estimates (total, mean, proportion as relevant) were arrived at for each setting. Based on the study objectives and keeping in mind the limited sample size (a total of 50 food retail outlets out of a total of 990, in the six selected sites), non-parametric comparative statistical analyses were limited to between countries. Independent samples Kruskal–Wallis test with Dunn’s post hoc pairwise comparison including Bonferroni correction was used to compare price of food items as well as fruit and vegetable diversity between countries. Though all the data on prices of food items was used in the comparative statistical analysis, the supplementary table (Table S3) shows the cheapest per food item type by food retail outlet and site, i.e., it is not an average price from the two outlets per site. To compare availability of food items across the three countries, Fisher’s Exact Test was used with adjusted residual post-hoc testing. For the total of all three countries together, Spearman’s correlation coefficient was used to assess correlation between in-community health and product promotion. Levels of significance for all tests were kept at *p* < 0.05. Where price estimations and comparisons were carried out, local costs or currencies were converted to international dollars using purchasing power parity exchange rates to improve the comparability of results. The exchange rates for 2017 were available for South Africa and Sweden through the Organisation for Economic Co-operation and Development’s (OECD) website [32]. The local currency was divided by the exchange rate (6.060 and 9.125 for South Africa and Sweden respectively), resulting in the International Dollar value. The 2017 exchange rate was not available for Uganda, so the 2016 exchange rate from the World Bank was used [33]. The inflation value from 2016 to 2017 was 2.1% for UGX [32]. The UGX rate was divided by the 2016 exchange rate (1098), and 2.1% of that was added in order to bring it to a comparable 2017 value.

### 2.4. Ethical Considerations

Ethical approval to conduct all research activities was sought and granted through the Makerere University (Uganda), University of the Western Cape (Cape Town, South Africa) and the Regional Ethics Review Board in Stockholm (Sweden). In Sweden the study was approved as part of the overall SMART2D project and trial (2015/712-31/1 and 2016/2521-31/1), and no further application was sought as the data collection activities were observational in nature, with no data collection involving participants and hence not required by law. Letters of ethical approval from the respective ethical review boards in each country were carried by the data collectors. During the ‘Food Retail Outlet Assessments’ the store owner and (or) manager were approached beforehand for permission to conduct the observations.

## 3. Results

**Figure 1:**
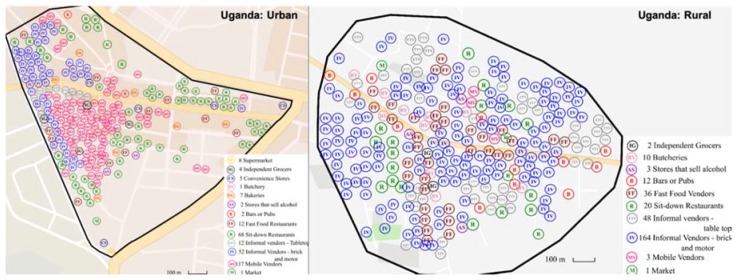
Distribution of food retail outlets in Uganda (not to scale).

**Figure 2:**
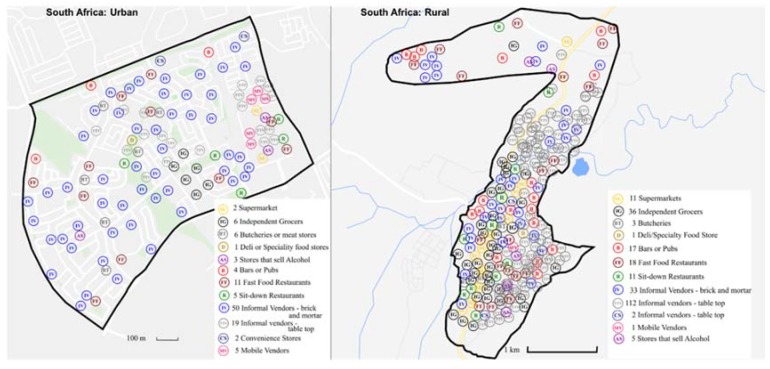
Distribution of food retail outlets in South Africa (not to scale).

**Figure 3:**
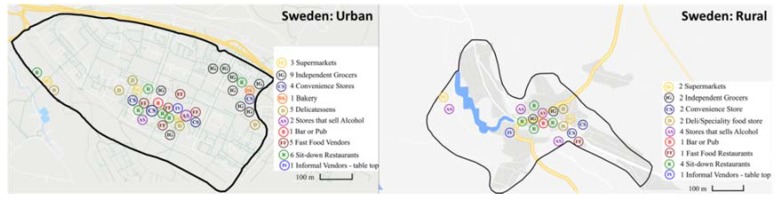
Distribution of food retail outlets in Sweden (not to scale).

Results from the food environment assessments are described below according to the external food environment domains as outlined and defined in the ANH-FEWG food environment conceptual framework [3], i.e., availability, price, vendor and product properties, as well as marketing and regulation. The main results showing between country comparisons are included in the text and additional details are presented in supplementary tables (Tables S1–S6). To avoid interpretation errors due to the small sample size, the highest and lowest counts are presented next to the means in each table. The variables are also presented by the different store types observed in the setting, by type of area (urban/rural) and as country totals. Additionally, for each site, an overview map was created to show the spread of food retail outlets (Figure 1, Figure 2 and Figure 3).

### 3.1. Availability

#### 3.1.1. Presence and Distribution of Food Retail Outlets

In the six selected sites, a total of 990 food retail outlets were identified during the overall mapping (excluding shops that sell alcohol) (Table 1). The most striking between-country differences relate to the overall number of outlets and food retail outlet type in the selected sites. Sweden had the fewest outlets of all three countries, with only 50 food retail outlets in total compared to 354 and 586 noted in South Africa and Uganda respectively. The spread of the food retail outlets is shown in Figure 1, Figure 2 and Figure 3 which have been resized to fit the text (not to scale). Maps to scale are available in Supplementary file S2, Figures S1–S6.

When considering presence of food retail outlet types, the most prominent difference between the countries was a high presence of informal food retail outlets in both Uganda and South Africa and a more formal food environment in Sweden (Table 1). Out of all three countries, the informal outlets played the biggest part in the food environment in Uganda (404), with markets containing many vendors noted in both urban and rural settings, a large number of mobile vendors (117) in the urban setting, as well as informal vendors (282). South Africa had large numbers of informal vendors (214) but just a few mobile vendors and no markets. In Sweden there was only a negligible presence of informal food retail outlets (2). South Africa had a higher number of formal food retail outlets (59) compared to Sweden (22) and Uganda (14), with higher numbers of supermarkets (11) and independent grocers (36) in the rural setting. Uganda had the most fast food vendors (48), compared to South Africa (29) and Sweden (6); and the rural setting had three times as many fast food vendors (36) compared to the urban setting (12). The pattern was similar in South Africa, but with smaller numbers, while in Sweden the fast food outlets were almost negligible. With respect to pubs or bars South Africa had the most (21), followed by Uganda (14) and Sweden (2). Notably, there were more than four times as many pubs or bars in the rural settings when compared to urban counterparts for South Africa and Uganda.

#### 3.1.2. Presence of Food Items in Food Retail Outlets

We carried out the ‘Food Retail Outlet Assessments’ in 50 food retail outlets across the three countries. The availability of food items varied by country as well as by urban or rural setting. On average, 47% of the food items were available in Uganda, compared to 66% in South Africa and 80% in Sweden (Table 2). When looking closer at the different food categories, the same pattern emerged – Sweden had the highest presence of food items and Uganda the lowest, with the rural setting in Uganda having the lowest average (35%). Findings from the Ugandan site reflected to some extent those from the South African site in that more of the food items were made available by retail outlets in the urban setting when compared to the rural setting; the opposite was true for Sweden. Confectionaries and sugar-sweetened beverages had a high presence across all study sites except for rural Uganda; in the latter site, confectionaries (19%) were less present than all the other foods and sweetened beverages were more available than other foods (52%) (Table 2). Confectionaries (biscuits, chips and chocolate bars) had a significantly higher presence in Sweden compared to Uganda but the differences with South Africa were not significant (Table 2). Overall, supermarkets in all the sites offered the most food options when compared to the other observed food retail outlet types.

With regard to the variety of fruits and vegetables offered, supermarkets played a notable role in providing a larger selection of fresh fruits and vegetables in all the countries. Additionally, open air markets in Uganda and the informal vendors in Sweden offered the greatest variety of fruits and vegetables (Tables S1 and S2). Mobile and informal vendors in Uganda and South Africa sold a limited range of fruits or vegetables per vendor. Frozen fruit was only available in Sweden and only present in supermarkets (not shown in table), while frozen vegetables were present in both supermarkets and independent grocers in Sweden and in supermarkets in South Africa (not shown in table).

### 3.2. Vendor and Product Properties

#### 3.2.1. Vendor Typology

Based on the vendor typologies described in the methods, there were some variations between individual outlets as well as between country differences regarding store content. The supermarkets that were assessed across all sites sold all the items listed in Table 2, while independent grocers sold a selection of the same. Convenience stores primarily sold ‘unhealthy’ food items and a small selection of other items, which in some cases included fruits or vegetables. Informal vendors were characterized by mainly selling fruits and/or vegetables with most specializing in a few types, apart from the one informal vendor in the urban Swedish site with a high variety. Mobile vendors specialized in 1–2 types of fruits or vegetables but did not sell any of the other listed items. Of the two markets that were assessed, both sold a large number of fruits and vegetables, as well as some other items.

#### 3.2.2. Product and Food Quality

We looked at quality mainly in terms of damaged fruits and vegetables. Supermarkets observed in Sweden had the least amount of damaged fruits when compared to supermarkets observed in the other two sites (Table S1). In the three countries, damaged fruits and vegetables were seen most often in the retail outlets that had the largest variety, except for supermarkets in Sweden (Tables S1 and S2).

When considering the ‘other’ grocery items such as breakfast cereal (low-, medium- or high in sugar, low-, source of or high-fiber), bread (wholemeal or white), milk (full cream or reduced fat) and yoghurt (plain or sweetened), these varied by country and item (Table S3). With respect to bread, though wholemeal was present in all three settings, there was a variation when compared to the presence of white bread. The majority of breads assessed in South Africa were wholemeal, in Sweden approximately half and very few in Uganda. In South Africa and Uganda, all but one of the observed milk was full cream, while in Sweden all but one was low or reduced fat. The observed yoghurt packs sold in South Africa and Uganda were sweetened and came in smaller containers, whereas in Sweden, they were plain (unsweetened) except for one, and came in 1 L packages. Regarding breakfast cereal across the countries, the majority were healthier alternatives—medium or low in sugar and high in fiber (oatmeal featured regularly).

### 3.3. Price

Overall, supermarkets and independent grocers offered the majority of items with the lowest prices across the three countries (Table S3). However, in general, supermarkets offered the lowest prices in urban sites and independent grocers in rural sites. In the rural Ugandan site, the lowest prices for fruits and vegetables were from informal vendors and markets respectively. Fruit and vegetables, as well as ‘other’ grocery items, were generally found at the lowest rates at supermarkets compared to confectionaries and beverages with the lowest prices found at independent grocers. When comparing prices, bread was significantly more expensive per package in Uganda than in Sweden, while the price per package of chips and biscuits was significantly lower in both South Africa and Uganda compared to Sweden (Table S3). Energy drinks in Uganda were significantly more expensive than in Sweden. In South Africa, packages of yoghurt had a significantly lower price than in Sweden. There were no significant differences between Uganda and South Africa with regard to prices of the other food items. There were no significant differences in fruit and vegetable price between countries.

### 3.4. Marketing and Regulation

#### 3.4.1. Advertising and Promotion

Overall, observed advertisements in all communities promoting unhealthy foods, drinks, and tobacco products (439) vastly outnumbered those promoting healthy lifestyle options (29) (Table 3; Table 4). In fact, advertisements promoting healthy lifestyle choices were not present in Uganda, minimal in South Africa (3) and more frequent in Sweden (26) (Table 3). This relationship was reverse for advertisements promoting unhealthy foods, drinks, and tobacco products (Uganda: 247; South Africa: 111; Sweden: 81); the rural site in Uganda had by far the highest number of advertisements for sweetened beverages (170) (Table 4). There was a strong negative correlation between country totals of product promotion and health promotion, which was statistically significant (*r_s_* = −0.880; *p* = 0.021) (Table 3 and Table 4), i.e., the lower the product promotion, the higher the health promotion in the setting. However, when observed, advertisements promoting a healthy diet (primarily found in Sweden) were overwhelmingly commercial in nature (Table 3). Tobacco product advertising in the community was low across all three study settings.

Supermarkets had most in-store advertisements for the most product categories, followed by convenience stores and small independent grocers—mobile vendors, informal vendors and markets had a negligible number (not in table). When looking specifically at junk food and sweetened beverages, which had the largest number of advertisements, supermarkets had the most, followed by independent grocers and then convenience stores (not in table). The retail outlets in Sweden had most advertisements for tobacco products compared to the other two countries. Sweden also had more in-store junk food advertisements than South Africa and significantly more compared to Uganda. For sweetened beverages there was no significant difference between countries. There was only a negligible number of fruits and vegetable advertisements overall, the majority in South Africa.

#### 3.4.2. Product Labelling

Overall, most of the food items other than fruits and vegetables were sold in packages (338 packaged out of 341 items). When considering food packaging, all three countries provided back-of-pack labels on product packages: nutrition information in the required language (91–100%); an ingredients list (77–92%); and a nutrition facts table or list (68–97%) (Table 5). With respect to front-of-pack labels, the presence of consumer guidance information such as guideline daily amounts or the Swedish keyhole symbol were seen in just under half of the Swedish products, around 20% of the South African products and 10% of the Ugandan products. Nutrition claims were most common among the South African products, followed by Uganda and then Sweden. Sweden had the lowest levels of health claims on the products and Uganda had the highest. There were urban–rural and outlet-level differences observed in product labelling, with the most variation seen in the front-of-pack labels (Tables S4–S6).

## 4. Discussion

This study presents snapshots of urban and rural food environments in three countries in different income groups. We identified three key findings through the use of the ANH framework: (1) Formal food retail outlets played a significant role in the provision of basic food items (fruits, vegetables, cereals, etc.) in all settings, however they were also the main contributors to the high presence of unhealthy food items (confectionaries and sweetened beverages); (2) Combination of formal and informal food outlets that contributed to the respective food environments varied in the three settings; the Ugandan setting had predominantly informal outlets, the South African setting had a more even combination of formal and informal, while the Swedish setting had primarily formal outlets; (3) Promotion of unhealthy products (junk food and sweetened beverages) by way of in-store and in-community advertisements was high in all settings and food labels on packaging provided back-of-pack information to a higher degree than front-of-pack labelling.

The overall availability of all food items was observed to correspond to the income status of the respective countries, with the Swedish setting having the highest and the Ugandan setting the lowest availability of observed items. Previous studies from the three countries show how nutritious diets are more expensive than less healthy diets [34,35,36]. Surprisingly, we found that for bread and breakfast cereal, healthier versions were often lower in price than the less healthy version. A recent systematic review looking at food prices globally found that, fruits and vegetables were moderately expensive in HICs and MICs and relatively expensive in LICs, when looking at caloric comparison [37]. There were no significant differences in price of fruits and vegetables between the countries in our study. The observed supermarkets offered most items and the lowest prices on fruits, vegetables and per package price of ‘other’ grocery items. A study from the US found that consumers sourced most food items from supermarkets, which was also the main source of junk food that was consumed [38]. In our study, the formal food retail outlets played an important role in making less healthy food options available in the study sites. Sweetened beverages and confectionaries had permeated all the study sites and were widely available compared to fruits and vegetables—with the exception of rural Uganda (which did not have a supermarket or convenience store). The high presence of processed foods and drinks is confirmed in studies across these countries. Moreover, consumption of sweetened beverages and other processed foods are among those that have increased most in the last few decades, with a simultaneous rise in obesity [39,40,41] and NCDs like type 2 diabetes [20,42]. This also links to recent findings of how obesity in rural areas is the main contributor to overall obesity worldwide, challenging the idea that urbanization is the main driver of obesity [43].

The results from the mapping tie in to how food environments globally have been conceptualized into three types—traditional, mixed and modern food systems [44]. The large number of informal and fast food outlets in Uganda and South Africa compared to Sweden in this study confirms how the informal food sector plays a key role in supplying urban and rural areas in sub-Saharan Africa [45,46]. From our results, one can broadly state that Uganda, particularly rural Uganda with a high reliance on informal food retail outlets may belong to the traditional food system type, leaning towards the mixed as the urban area had more formal outlets. South Africa, with the prominent presence of both informal and formal food retail outlets found in this study nests well in the mixed food system type [44,47]. Street foods are a benchmark of the mixed food system type [44], something that one could see clearly both in the Ugandan and South African setting where many of the fast food vendors sell popular traditional and new dishes and snacks, like the rolled chapatti bean omelet known as ‘Rolex’ [48]. Sweden’s food environment is highly regulated [49], which may explain the limited number of informal outlets observed in this setting; comprised primarily of formal food retail outlets which could be considered a modern type food system [44,47]. Some have argued that the introduction of supermarkets has been positive due to their ability to offer lower food prices and bring in fresher and safer food options [50], while others have pointed out that the larger unit sizes offered may be unaffordable for the poorest, and that these outlets are often situated in inconvenient locations in lower-income areas [51,52]. The latter was not supported in this study as all sites in this study contained food retail outlets (including supermarkets) within a relatively small radius, particularly in urban areas, though the mapping did not include distances from homes to food retail outlets. On the other hand, the informal food sector can be present in spots convenient to buyers and may be more adaptable to local consumers’ needs, selling more affordable products and in smaller quantities with the added possibility of buying on credit. They can therefore be essential for vulnerable residents [45] and potentially play a more prominent role in improving the health of the populations they serve [45,53,54,55]. In this study however, we did not find that informal food retail outlets had lower prices per package on the items observed, though this may have been different if other food items were considered. In a bid to modernize and ‘tidy up’ cities, governments have put restrictions on the informal food sector and have prioritized more formal outlets like supermarkets [45]; informal vendors face constant harassment, forced relocation, bribes and general marginalization [55,56].

In this conceptualized traditional, mixed and modern food system, both product and health promotion is thought to increase for each step towards the modern food system [44]. Interestingly, our findings were the exact opposite for (in-community) product promotion, with Uganda (traditional-mixed) having the most, which may be explained by differences in regulation between the countries. However, health promotion (in-community) followed the traditional to modern trajectory with Sweden having the most [44]. It is also interesting to note that health promotion was commercial in nature, indicating that it could be considered proxy product promotion. With regard to in-store advertisements we found that the formal food retail outlets played the main role in promoting unhealthy products, while informal outlets had negligible advertising. Particularly for the LMICS, it has been postulated that having products as well as advertisements in communities that otherwise may not have access to these food items can potentially accelerate the nutrition transition [52]. Food packaging can been considered as a form of product promotion and this includes back-of-pack and front-of-pack labels [57]. A study focusing on biscuit and chip packages from 16 countries found that 86% of the packages had nutrition labels and 30% had nutrition or health claims [58], which was similar to our study. Though we had a broader range of products, we saw similar high levels of back-of-pack (nutrition) labels and fewer front-of-pack labels including nutrition and health claims (5–42%). Interestingly, Uganda had the highest level of health claims, while Sweden had the least number of health and nutrition claims. Studies have shown that consumers find both back-of-pack and front-of-pack labelling confusing, though the function of a label is to help the consumer [57,59]. There is a risk that nutrition and health claims can mislead consumers by focusing on one nutrient as positive when the rest of the product may not be conducive to health. Differences in front-of-pack labels between the countries may relate to differences in regulations and their implementation. Consumer guidance information was highest in Sweden and lowest in Uganda. The level of consumer guidance information fits into the food systems typologies that suggests traditional food systems as having low levels of information and modern food systems having higher levels [44]. The consumer guidance information for modern type in this study, was primarily the industry-led guideline daily amounts (GDA), and the ‘Keyhole’ symbol. When comparing types of front-of-pack consumer guidance labelling schemes, those using traffic lights or symbols that show an overall rating of a product like the Swedish ‘Keyhole’, have been found successful [60,61,62]; another recent development in this direction, is the ‘Nutri-Score’, which combines a five-color code system with a letter rating [62].

### 4.1. Implications for NCD Prevention and Interventions

This study identifies relevant contextual differences between the food environments in the selected sites from a prevention and intervention standpoint. Understanding the context in which interventions are developed and implemented is crucial and the physical environment is an integral aspect of the context [63]. When considering issues of accessibility to healthy foods as part of downstream interventions [64] for the prevention of diet-related NCDs, there is a need for context-specific pointers to enable populations to proficiently navigate their food retail environments. For example: where can participants access healthier food options at the most affordable prices (formal vs. informal vendors; supermarkets vs. convenience stores; etc.); how to overcome issues related to the availability (or lack thereof) of vendors stocking healthier food products (ride-sharing initiatives or co-ops among participants; creation and maintenance of a community or household garden; etc.); in-store demonstration or training to read nutritional information on packaged products (what ingredients to look for; how to read nutritional information; how to be savvy to health and nutrition claims made on packaging; etc.); how to navigate supermarket aisles with a better understanding of in-store advertising and product placement.

The nature of the findings ultimately also speaks to the need for more effective upstream and mid-stream interventions that would address key food environment-related factors on a larger scale. To improve health outcomes, policy measures and regulations such as taxation, e.g., sugar tax [65] should be combined with behaviour-change strategies taking into consideration the food environment and individual level factors in order to decrease consumption [66]. Subsidizing healthier options, especially for more vulnerable groups could increase consumption by making these foods more accessible [65,67,68]. As part of urban planning, zoning laws can determine what type of outlets are allowed to set up business, and may thus help change the ratio of outlets that sell healthier options compared to those selling processed foods with low nutritional value [69]. Regulations on food labelling can nudge food companies to reformulate or create healthier food products through nutrient profiling and indirectly through increased consumer demand for marked products [70]. In addition, working with local retail outlets to nudge customers towards healthier options and implementing worksite- [71] or school-based interventions [72] to promote healthy food behaviours have been found to be promising. Stronger government policies and local actions that make food environments more conducive to healthy choices through restricted advertising of unhealthy food and beverages, as well as regulated and user-friendly food labelling, could go a long way in changing the foodscapes in these settings, and ultimately in reducing diet-related NCDs [73,74,75,76,77].

### 4.2. Study Strengths and Weaknesses

The strengths of this study include the use of a tool that was assessed for reliability in multiple sites [29], and further modified to capture additional details relating to NCDs like type 2 diabetes that share diet as a common risk factor. Taking an NCD perspective when adapting the EPOCH tool provided a clear framework to map external food environments in the selected sites. The ‘snapshots’ taken using the EPOCH tool aimed to be representative of each study site. Issues relating to the classification of stores and products were pre-empted as much as possible by consulting with local, in-country partners—with an eye towards maximum consistency across the three settings. Study settings were identified as being under-resourced (South Africa and Uganda) or socio-economically disadvantaged (Sweden) and these findings, while not generalizable, may be transferable to areas with similar population characteristics. Being part of a multi-center trial meant that local experts from each of the countries were involved in the study, allowing for more context specific detail in the data, thus strengthening the study.

However, there are some limitations to the study, relating to the sample and cross-sectional design. The food mapping consisted of two sites per country (one urban and rural each), resulting in a ‘snapshot’. Though the sites were chosen to match certain characteristics, they were spread across different area sizes, had varying populations, income levels, and demographics, and were not selected to be representative of other areas in each country. The study was cross sectional in nature and the day of the week or season when data collection took place could have influenced inferences on availability—both on the retail outlet and product level. Using the mapping tool, the pre-determined options of store typologies had to be filled and meant that the outlets visited in the three countries had to fit into those particular typologies. Certain food categories within the tool were not consumed equally as part of the local diets in the three countries, which may make price and availability comparisons misleading. This was rectified by focusing on those that were common such as fruits, vegetables, confectionaries and sweetened drinks.

## 5. Conclusions

The food environment has an important role in population health through influencing food choices and making foods available. The three study settings represent the concept of traditional (low income: Uganda), mixed (middle income: South Africa) and modern (high income: Sweden) food systems, illustrated by the decreasing prominence of informal food retail outlets compared to formal outlets. Formal food retail outlets were found to be a double-edged sword –both providing a variety of foods at lower prices, as well as advertising and making unhealthy food items readily available. In-community product advertising was by far highest in Uganda and lowest in Sweden, perhaps as a reflection of differences in regulation in the two settings. The findings speak to the need to address contextual differences in NCD-related health interventions by incorporating strategies that address food environments, and for a critical look at regulations that tackle key food environment-related factors on a larger scale.

## Figures and Tables

**Table 1:** Food retail outlets in selected sites in Uganda, South Africa and Sweden.

		Uganda		South Africa			Sweden		
Food Retail Outlet Type	Urban n	Rural n	Total n	Urban n	Rural n	Total n	Urban n	Rural n	Total n
Formal food retail outlets									
Supermarket	3	0	3	2	11	13	3	2	5
Independent grocer	4	2	6	6	36	42	9	2	11
Convenience store	5	0	5	2	2	4	4	2	6
Total	12	2	14	10	49	59	16	6	22
Stores with specialty products									
Butcher/meat store	1	10	11	6	3	9	0	0	0
Bakery	7	0	7	0	0	0	1	0	1
Deli/specialty food store	0	0	0	1	1	2	5	2	7
(Stores that sell alcohol)	2	3	5	3	5	8	2	4	6
Total	10	13	23	10	9	19	8	6	14
Food service outlets									
Pubs/Bars	2	12	14	4	17	21	1	1	2
Fast food vendors	12	36	48	11	18	29	5	1	6
Other sit-down restaurants	68	20	88	3	11	14	6	4	10
Total	82	68	150	18	46	64	12	6	18
Informal food retail outlets									
Informal vendor—table top	18	48	66	19	112	131	1	1	2
Informal vendor—brick and mortar	52	164	216	50	33	83	0	0	0
Mobile vendor	117	3	120	5	1	6	0	0	0
Market	1	1	2	0	0	0	0	0	0
Total	188	216	404	74	146	220	1	1	2
Overall Total ^†^	290	296	586	109	245	354	35	15	50

^†^ excluding stores that sell alcohol to avoid double counting.

**Table 2:** Availability of food items in retail outlets by country (urban/rural).

Food Items	Uganda Urban 11; Rural 7; Total 18			South Africa Urban 8; Rural 11; Total 19			Sweden Urban 7; Rural 6; Total 13		
	Urban n (%) †	Rural n (%)	Total n (%)	Urban n (%)	Rural n (%)	Total n (%)	Urban n (%)	Rural n (%)	Total n (%)
Fruits	8 (72.7)	2 (28.6)	10 (55.6)	5 (62.5)	7 (63.6)	12 (63.2)	4 (57.1)	5 (83.3)	9 (69.2)
Vegetables	7 (63.6)	3 (42.9)	10 (55.6)	5 (62.5)	6 (54.5)	11 (57.9)	4 (57.1)	5 (83.3)	9 (69.2)
Mean n and %	7.5 (68.2)	2.5 (35.7)	10 (55.6)	5 (62.5)	6.5 (59.1)	11.5 (60.5)	4 (57.1)	5 (83.3)	9 (69.2)
Other groceries:									
Breakfast cereal	5 (45.5)	1 (14.3)	6 (33.3)	7 (87.5)	6 (54.5)	13 (68.4)	4 (57.1)	5 (83.3)	9 (69.2)
Bread	4 (36.4)	4 (57.1)	8 (44.4)	6 (75)	5 (45.5)	11 (57.9)	4 (57.1)	6 (100)	10 (76.9)
Milk	5 (45.5)	2 (28.6)	7 (38.9)	7 (87.5)	6 (54.5)	13 (68.4)	4 (57.1)	4 (66.7)	8 (61.5)
Yoghurt	5 (45.5)	2 (28.6)	7 (38.9)	7 (87.5)	3 (27.3)	10 (52.6)	3 (42.9)	6 (100)	9 (69.2)
Mean n and %	4.8 (43.2)	2.3 (32.1)	7 (38.9)	6.8 (84.4)	5 (45.5)	11.8 (61.8)	3.8 (53.6)	5.3 (87.5)	9 (69.2)
Confectionaries:									
Biscuits	6 (54.5)	3 (42.9)	9 (50) *^.^‡	7 (87.5)	7 (63.6)	14 (73.7) *	6 (85.7)	6 (100)	12 (92.3) *
Chips	5 (45.5)	0 (0)	5 (27.8) *	7 (87.5)	8 (72.7)	15 (78.9) *	6 (85.7)	6 (100)	12 (92.3) *
Chocolate bar	5 (45.5)	1 (14.3)	6 (33.3) *	7 (87.5)	5 (45.5)	12 (63.2) *	6 (85.7)	5 (83.3)	11 (84.6) *
Mean n and %	5.3 (48.5)	1.3 (19)	6.7 (37)	7 (87.5)	8 (72.7)	13.7 (71.9)	6 (85.7)	5.7 (94.4)	11.7 (89.7)
Sweetened beverages:									
Non-diet soda	6 (54.5)	4 (57.1)	10 (55.6)	7 (87.5)	6 (54.5)	13 (68.4)	6 (85.7)	6 (100)	12 (92.3)
Fruit drink	7 (63.6)	3 (42.9)	10 (55.6)	7 (87.5)	6 (54.5)	13 (68.4)	6 (85.7)	6 (100)	12 (92.3)
Energy drink	6 (54.5)	4 (57.1)	10 (55.6)	7 (87.5)	6 (54.5)	13 (68.4)	6 (85.7)	6 (100)	12 (92.3)
Mean n and %	6.3 (57.6)	3.7 (52.4)	10 (55.6)	7 (87.5)	6 (54.5)	13 (68.4)	6 (85.7)	6 (100)	12 (92.3)
Overall mean n and %	6.0 (54.4)	2.4 (34.8)	8.4 (46.8)	6.4 (80.5)	6.4 (58.0)	12.5 (65.7)	4.9 (70.5)	5.5 (91.3)	10.4 (80.1)

† % denotes the proportion of urban, rural or total number of stores as applicable for each column. ‡ *p*-values based on Fisher’s Exact Test to compare availability of food items across countries, with significance at * ≤0,05.

**Table 3:** Health promotion (advertising) in the community.

Type of Promotion	Uganda		South Africa		Sweden	
	Urban	Rural	Urban	Rural	Urban	Rural
Diet (non-commercial)	0	0	0	0	1	0
Diet (commercial)	0	0	0	1	6	10
Physical activity (non-commercial)	0	0	0	0	4	0
Physical activity (commercial)	0	0	0	0	1	1
Signs prohibiting smoking	0	0	0	0	2	1
Smoking cessation	0	0	0	2	0	0
Alcohol cessation	0	0	0	0	0	0
Total	0	0	0	3	14	12
Country total	0		3		26	

**Table 4:** Product promotion (advertising) in the community.

Type of Advertising	Uganda		South Africa		Sweden		
	Urban	Rural	Urban	Rural	Urban	Rural	Total
‘Junk food’	21	2	15	27	36	20	121
Sweetened beverages	27	170	30	8	10	4	249
Cigarette or tobacco product	0	0	1	0	1	1	3
Alcoholic drinks	6	21	15	15	2	7	66
Total	54	193	61	50	49	32	439
Country Total	247		111		81		

**Table 5:** Packaged food product labelling—country totals.

		Back-of-Pack Label			Front-of-Pack Label		
	Products with a Package	Nutrition Info in Required Language †	Ingredients List	Nutrition Facts	Consumer Guidance Info	Nutrition Claim	Health Claim
Urban							
Uganda	n = 57	53 (92.9%)	44 (77.2%)	43 (75.4%)	6 (10.5%)	9 (15.8%)	17 (29.8%)
South Africa	n = 76	69 (90.8%)	61 (80.3%)	61 (80.3%)	18 (23.7%)	32 (42.1%)	18 (23.7%)
Sweden	n = 55	52 (94.5%)	49 (89.1%)	51 (92.7%)	26 (47.2%)	10 (18.2%)	5 (9.1%)
Rural							
Uganda	n = 25	25 (100%)	23 (92.0%)	17 (68%)	2 (8%)	5 (20.0%)	6 (24.0%)
South Africa	n = 63	60 (95.2%)	53 (84.1%)	55 (87.3%)	12 (19.0%)	23 (36.5%)	13 (20.6%)
Sweden	n = 62	61 (98.4%)	56 (90.3%)	60 (96.7%)	28 (45.1%)	9 (14.5%)	3 (4.8%)

† The categories are not mutually exclusive.

## Supplementary Materials

The following are available online at https://www.mdpi.com/2072-6643/12/2/484/s1. Table S1: Fruits—diversity, quality, price by food retail outlet; Table S2: Vegetables—diversity, quality, price by food retail outlet; Table S3: Cheapest food items available by retail outlet type and country (urban, rural); Table S4: Packaged food product labelling—South Africa; Table S5: Packaged food product labelling—Sweden; Table S6: Packaged food product labelling—Uganda; Figure S1: Distribution of food retail outlets in urban Uganda; Figure S2: Distribution of food retail outlets in rural Uganda; Figure S3: Distribution of food retail outlets in urban South Africa; Figure S4: Distribution of food retail outlets in rural South Africa; Figure S5: Distribution of food retail outlets in urban Sweden; Figure S6: Distribution of food retail outlets in rural Sweden.
