# Supplementary material for: Snapshots of Urban and Rural Food Environments: EPOCH-Based Mapping in a High-, Middle-, and Low-Income Country from a Non-Communicable Disease Perspective
Source: Nutrients. 2020 Feb 14;12(2):484. doi: 10.3390/nu12020484 (PMC7071357; doi:10.3390/nu12020484)
Supplement: Supplementary file 1 [file nutrients-12-00484-s001.zip › Supplementary file S2.docx]

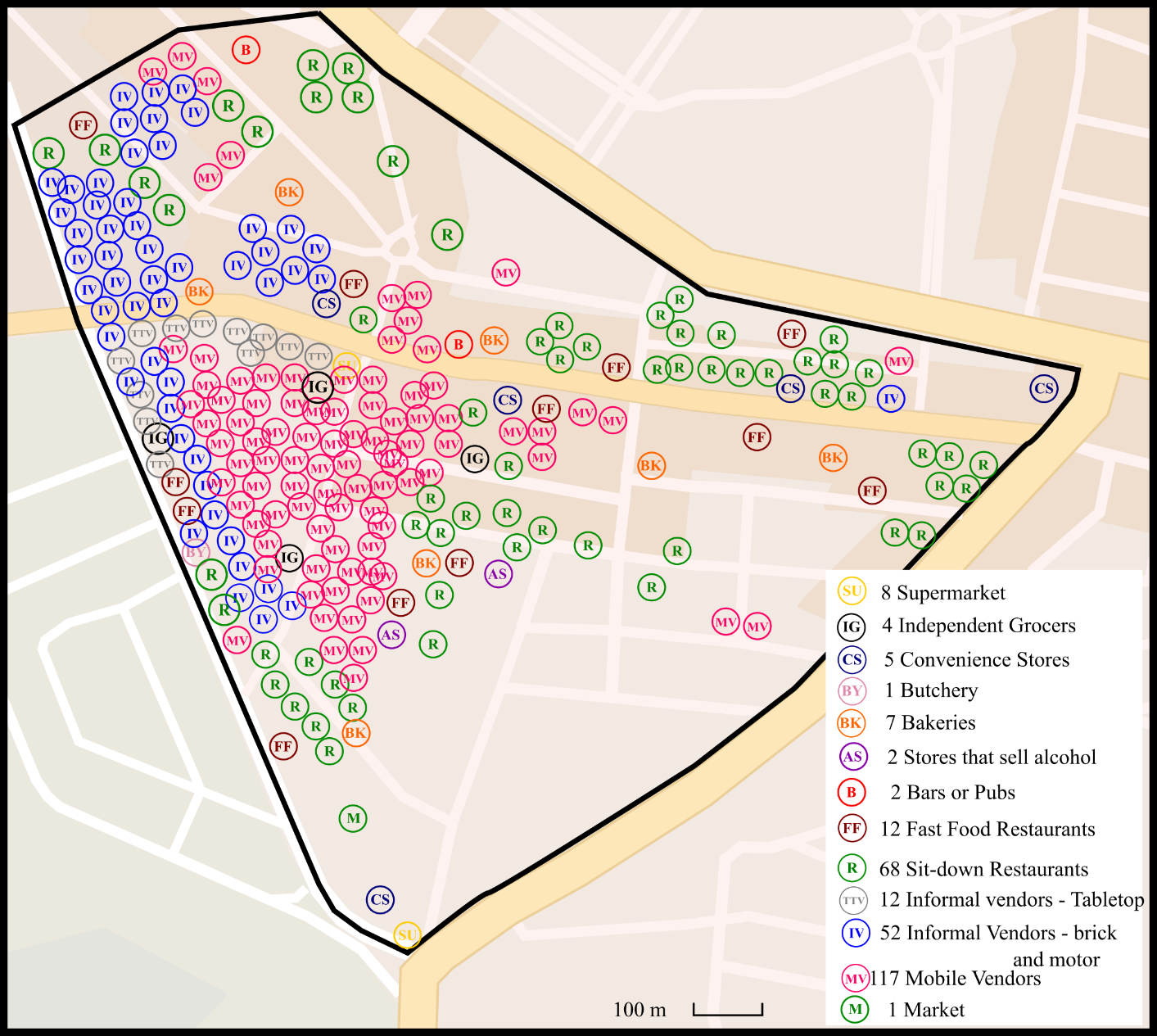


**UGANDA URBAN**

**Figure S1.** Distribution of food retail outlets in urban Uganda.


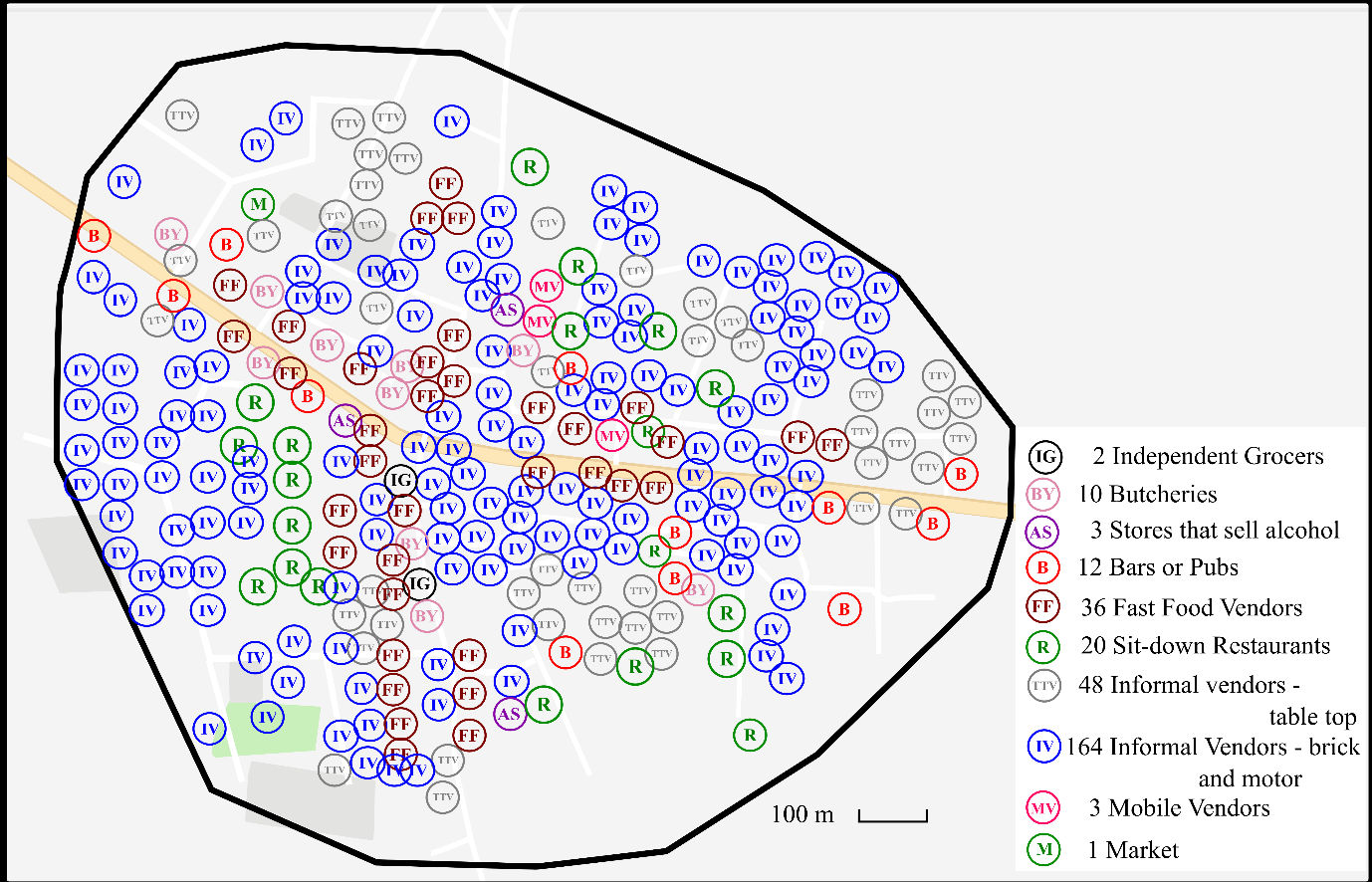
**Figure S2.** Distribution of food retail outlets in rural Uganda.

**UGANDA RURAL**


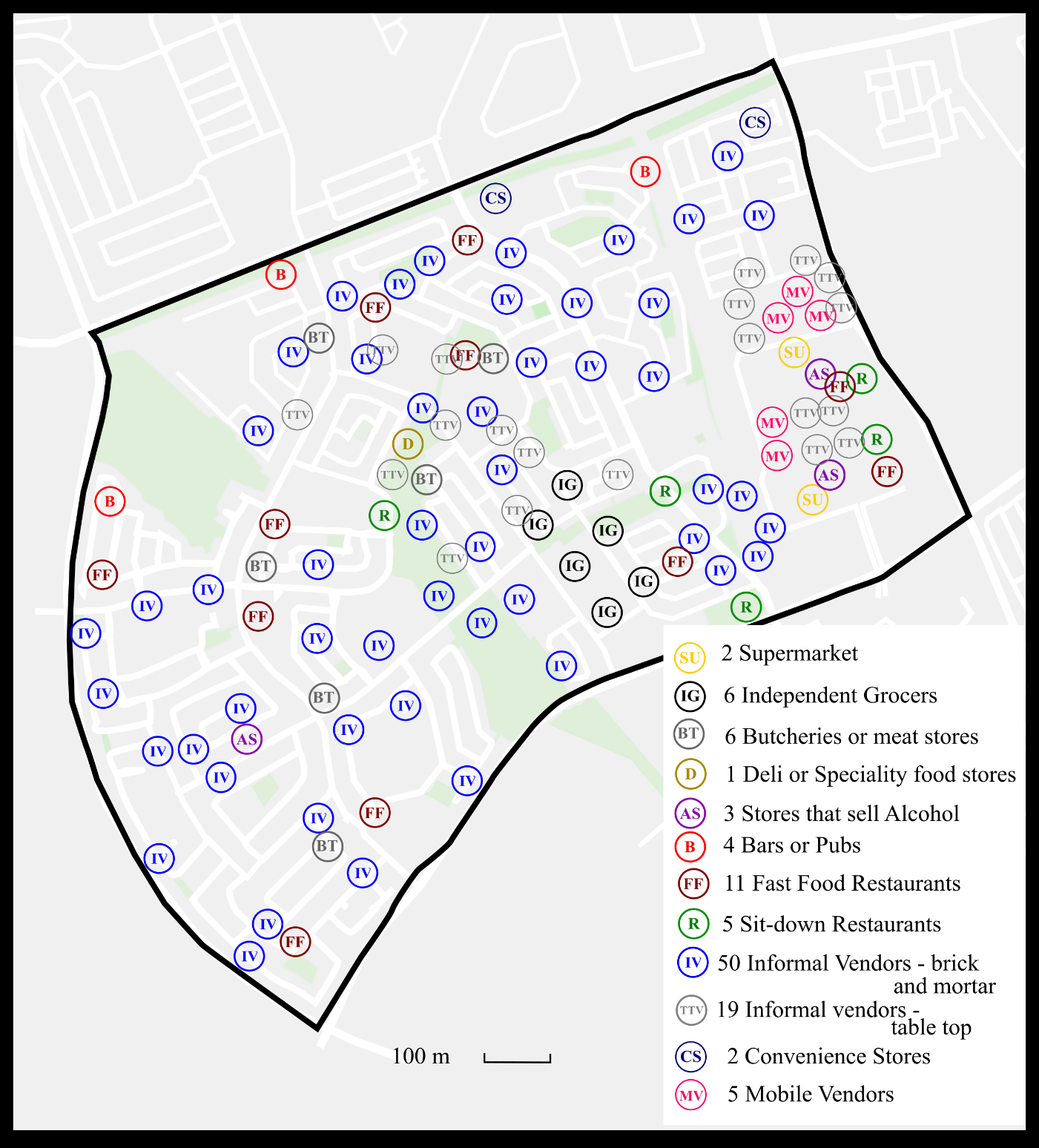


**SOUTH AFRICA URBAN**

**Figure S3.** Distribution of food retail outlets in urban South Africa.


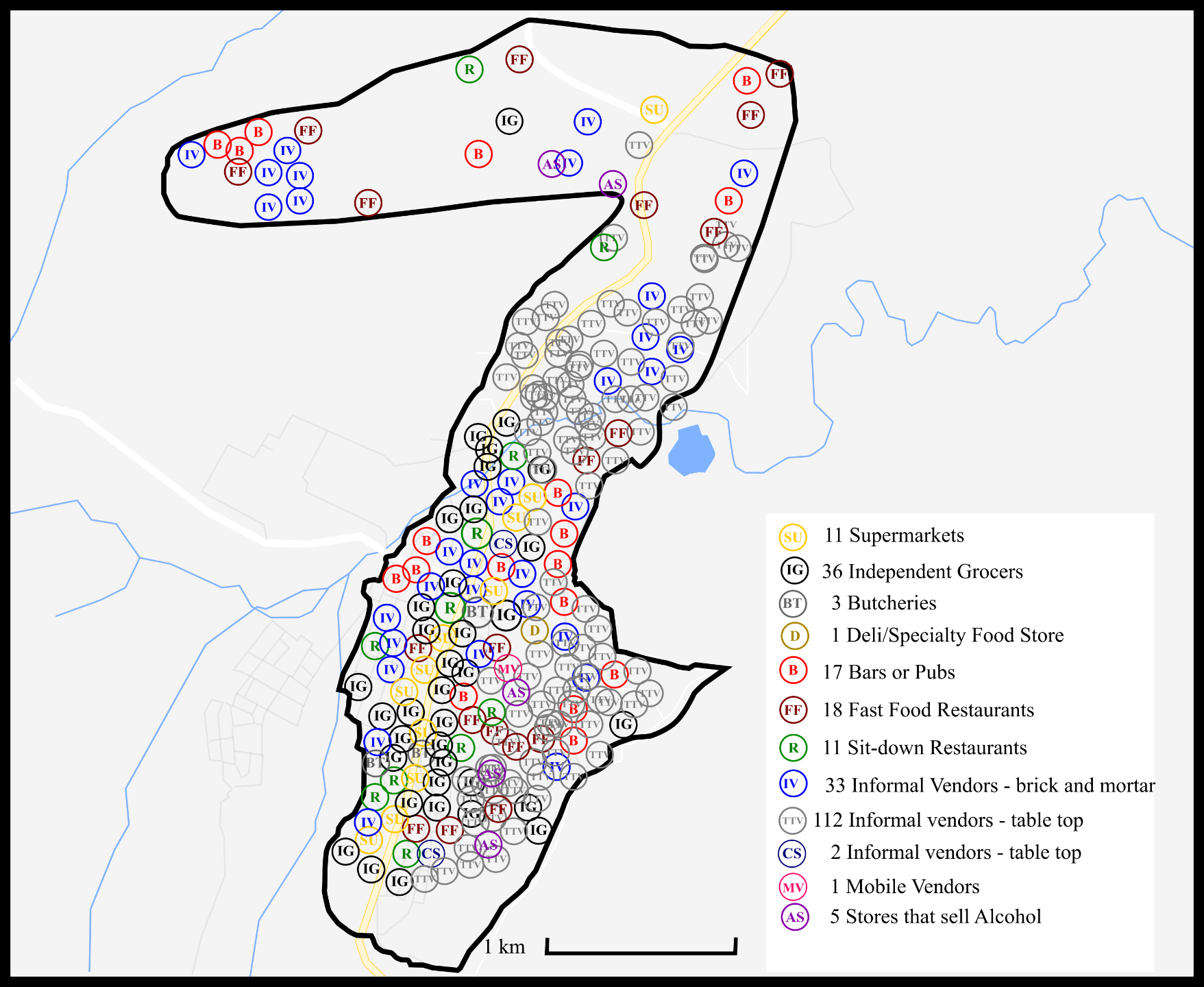


**SOUTH AFRICAL RURAL**

**Figure S4.** Distribution of food retail outlets in rural South Africa.


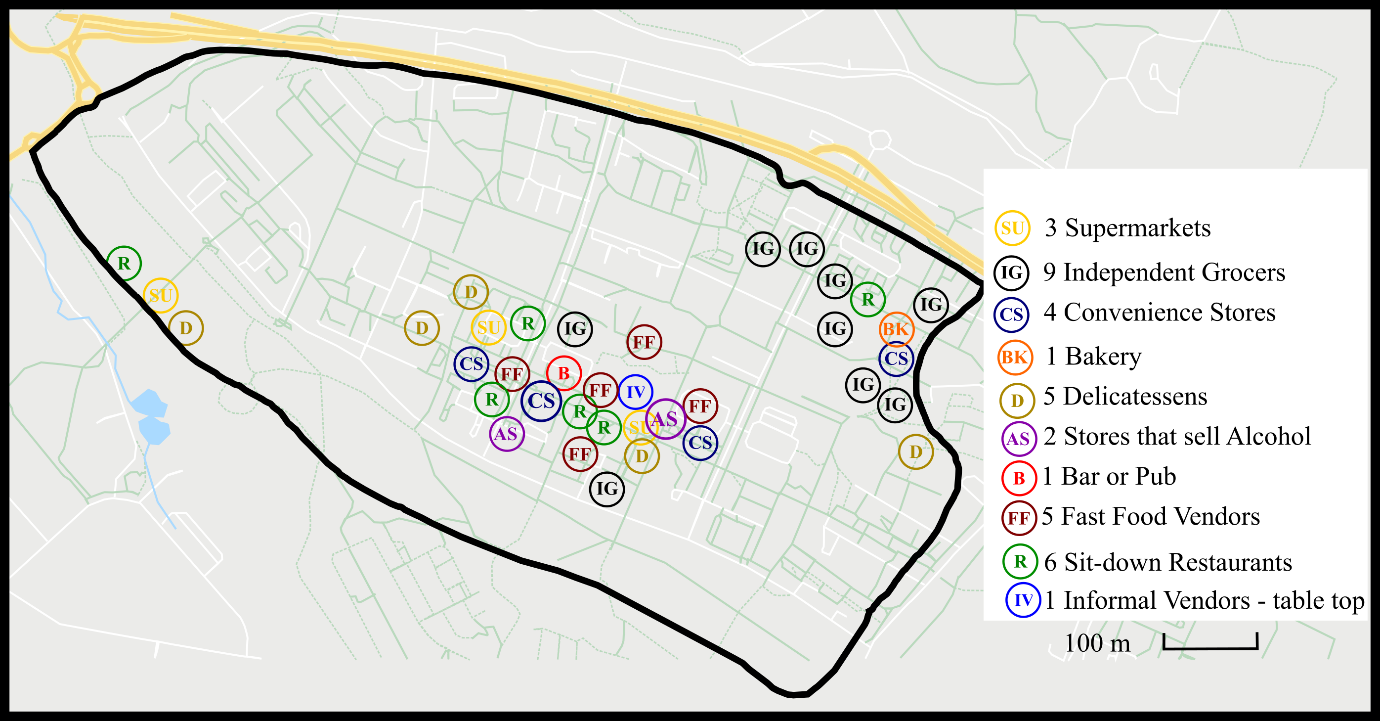


**SWEDEN URBAN**

**Figure S5.** Distribution of food retail outlets in urban Sweden.


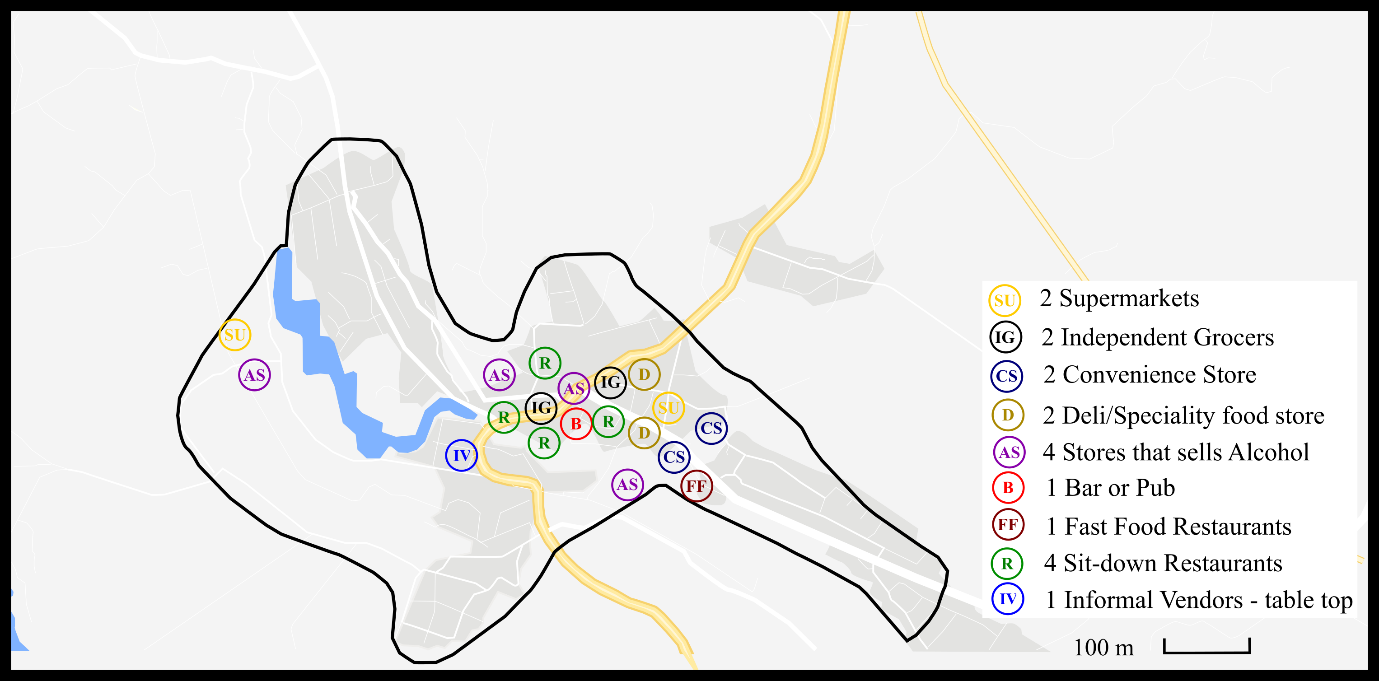


**SWEDEN RURAL**

**Figure S6.** Distribution of food retail outlets in rural Sweden.
