# Supplementary material for: Snapshots of Urban and Rural Food Environments: EPOCH-Based Mapping in a High-, Middle-, and Low-Income Country from a Non-Communicable Disease Perspective
Source: Nutrients. 2020 Feb 14;12(2):484. doi: 10.3390/nu12020484 (PMC7071357; doi:10.3390/nu12020484)
Supplement: Supplementary file 1 [file nutrients-12-00484-s001.zip › Supplementary file S1.docx]

| **Table S1:** Fruits –diversity, quality, price by food retail outlet | | | | | | | | | | | | | |
| --- | --- | --- | --- | --- | --- | --- | --- | --- | --- | --- | --- | --- | --- |
|  |  | **Supermarket** | | **Independent Grocer** | | **Convenience store** | | **Informal vendor** | | **Mobile vendor** | | **Market** | |
| **Urban, Rural** |  | **U** | **R** | **U** | **R** | **U** | **R** | **U** | **R** | **U** | **R** | **U** | **R** |
| **Total number of outlets mapped** | UG | 2 | 0 | 2 | 2 | 2 | 0 | 2 | 3 | 2 | 1 | 1 | 1 |
|  | SA | 2 | 2 | 3 | 2 | 2 | 2 | 2 | 2 | 0 | 2 | 0 | 0 |
|  | SW | 2 | 2 | 2 | 2 | 2 | 2 | 1 | 0 | 0 | 0 | 0 | 0 |
| **Fresh fruit availability, number of outlets** | UG | 2 |  | 0 | 0 | 1 |  | 2 | 1 | 2 | 0 | 1 | 1 |
|  | SA | 2 | 2 | 0 | 1 | 1 | 0 | 2 | 2 |  | 2 |  |  |
|  | SW | 2 | 2 | 1 | 2 | 0 | 1 | 1 |  |  |  |  |  |
| **Fruits visible from outside the shop, number of outlets** | UG | 0 |  | - | - | 1 |  | 2 | 1 | 2 | - | 1 | 1 |
|  | SA | 0 | 2 | - | 1 | 0 | - | 1 | 2 |  | 2 |  |  |
|  | SW | 0 | 0 | 0 | 0 | - | 0 | 1 |  |  |  |  |  |
| **More than 3 types of fruit appear to be damaged, number of outlets** | UG | 2 |  | - | - | 0 |  | 0 | 1 | 0 | - | 1 | 1 |
|  | SA | 2 | 2 | - | 0 | 0 | - | 0 | 2 |  | 0 |  |  |
|  | SW | 0 | 0 | 0 | 1 | - | 0 | 1 |  |  |  |  |  |
| **Average number of fruits** (min,max) | UG | 17 (13,21) |  | - | - | 1 (1) |  | 2 (2,2) | 3 (3) | 2 (2,2) | - | 27 (27) | 10 (10) |
|  | SA | 14 (9,19) | 12 (9,15) | - | 9 (9) | 7 (7) | - | 5,5 (4,7) | 2,5 (2,3) |  | 1,5 (1,2) |  |  |
|  | SW | 28,5 (26,31) | 25 (23,27) | 12 (12) | 16 (4,28) | - | 4 (4) | 48 (48) |  |  |  |  |  |
| **Citrus fruit** average price per kg (Intl.$) †  Types: SA: orange, lemon, satsuma; SW: orange, lemon, satsuma, clementine; UG: orange, lemon, tangerine | UG | 5,12 |  | - | - | - |  | 0,69 | 0,93 | 1,86 | - | 2,33 | 2,24 |
|  | SA | 0,91 | 1,65 | - | 0,99 | 1,65 | - | 1,49 | 4,13 |  | 6,6 |  |  |
|  | SW | 2,33 | 2,24 | 2,19 | 2,47 | - | 1,85 | 1,64 |  |  |  |  |  |
| **Non-citrus fruit** average price per kg (Intl.$)^1^ Types: SA: Apple, banana, pear; SW: banana, watermelon, melon, apple  UG: banana, apple, papaya, guava, avocado | UG | 2,88 |  | - | - | 7,81 |  | 1,86 | - | 3,72 | - | 1,40 | 1,86 |
|  | SA | 1,73 | 2,06 | - | 0,82 | 2,48 | - | 2,15 | 2,89 |  | 1,65 |  |  |
|  | SW | 0,82 | 2,30 | 2,08 | 1,81 | - | 3,28 | 1,09 |  |  |  |  |  |
| † Conversion rates: SA: /6.060; SW: /9.125; UG: /1098 (+2.1%)  - signifies unavailability of that item   \|  \| A black box indicates that the retail outlet is not present. \| \| --- \| --- \| | | | | | | | | | | | | | |

| **Table S2:** Vegetables –diversity, quality, price by food retail outlet | | | | | | | | | | | | | |
| --- | --- | --- | --- | --- | --- | --- | --- | --- | --- | --- | --- | --- | --- |
|  |  | **Supermarket** | | **Independent Grocer** | | **Convenience store** | | **Informal vendor** | | **Mobile vendor** | | **Market** | |
| Urban, Rural |  | **U** | **R** | **U** | **R** | **U** | **R** | **U** | **R** | **U** | **R** | **U** | **R** |
| **Total number of outlets mapped** | UG | 2 | 0 | 2 | 2 | 2 | 0 | 2 | 3 | 2 | 1 | 1 | 1 |
|  | SA | 2 | 2 | 3 | 2 | 2 | 2 | 2 | 2 | 0 | 2 | 0 | **0** |
|  | SW | 2 | 2 | 2 | 2 | 2 | 2 | 1 | 0 | 0 | 0 | 0 | 0 |
| **Fresh vegetable availability, number of outlets** | UG | 2 |  | 0 | 0 | 0 |  | 2 | 1 | 0 | 1 | 1 | 1 |
|  | SA | 2 | 2 | 0 | 3 | 1 | 0 | 2 | 1 |  | 0 |  |  |
|  | SW | 2 | 2 | 1 | 2 | 0 | 1 | 1 |  |  |  |  |  |
| **Vegetables visible from outside the shop, number of outlets** | UG | 0 |  | - | - | - |  | 2 | 1 | - | 1 | 1 | 1 |
|  | SA | 0 | 2 | - | 3 | 0 | - | 1 | 1 |  | - |  |  |
|  | SW | 0 | 0 | 0 | 0 | - | 0 | 1 |  |  |  |  |  |
| **More than 3 types of Vegetables appear to be damaged, number of outlets** | UG | 1 |  | - | - | - |  | 0 | 1 | - | 0 | 1 | 1 |
|  | SA | 1 | 2 | - | 0 | 0 | - | 0 | 0 |  | - |  |  |
|  | SW | 0 | 1 | 0 | 2 | - | 0 | 1 |  |  |  |  |  |
| **Average number of vegetables** (min,max) | UG | 12 (2,22) |  | - | - | - |  | 3 (1,5) | 5 (5) | - | 1 (1) | 65 (65) | 11 (11) |
|  | SA | 35,5 (24,47) | 27 (23,31) | - | 11 (4,25) | 14 (14) | - | 12,5 (12,13) | 1 |  | - |  |  |
|  | SW | 42,5 (41,44) | 42  (39, 45) | 5 (5) | 17,5 (12,23) | - | 5 (5) | 50 (50) |  |  |  |  |  |
| **Cruciferous veg** price per Kg (Intl.$)†  Types: SA: cabbage; SW: cabbage, cauliflower; UG: cabbage | UG | 0,47 |  | - | - | - |  | - | 0,47 | - | 0,93 | 1,86 | 0,56 |
|  | SA | 1,11 | 0,99 | - | 0,66 | 1,24 | - | 1,49 | - |  | - |  |  |
|  | SW | 0,87 | 0.98 | - | 1,75 | - |  | 0,77 |  |  |  |  |  |
| **Dark yellow** **vegetable** average price per kg (Intl.$)  Types: SA: carrot; SW: carrot; UG: sweet potato, carrot | UG | 1,01 |  | - | - | - |  | - | - | - | - | 1,40 | 2,33 |
|  | SA | 0,74 | 1,48 | - | 0,66 | 1,73 | - | 1,44 | - |  | - |  |  |
|  | SW | 1,36 | 1,28 | - | 1,21 | - | - | 1,10 |  |  |  |  |  |
| **Green leafy vegetable** average price per kg (Intl.$)  Types: SA: spinach, lettuce; SW: iceberg lettuce, Chinese cabbage; UG: ‘dodo’, ‘nakati’, ‘gobe’ | UG | 2,04 |  | - | - | - |  | - | - | - | - | 2,33 | 0,75 |
|  | SA | 1,54 | 2,56 | - | - | 3,63 | - | 3,96 | - |  | - |  |  |
| **Other vegetable** average price per kg (Intl.$)  Types: SA: onion, potato, beetroot, cucumber, corn; SW: onion; UG: eggplant, okra, ’ntula’, baby marrow | UG | 4,19 |  | - | - | - |  | 2,33 | 0,37 | - | - | 2,79 | 0,28 |
|  | SA | 1,02 | 2,80 | - | 0,80 | 1,72 | - | 1,82 | 2,48 |  | - |  |  |
|  | SW | 0,82 | 1,25 | 1,10 | 0,99 | - | 1,85 | 0,88 |  |  |  |  |  |
| † Conversion rates: SA: /6.060; SW: /9.125; UG: /1098 (+2.1%), - signifies unavailability of that item,   \|  \| A black box indicates that the retail outlet is not present. \| \| --- \| --- \| | | | | | | | | | | | | | |
|  | | | | | | | | | | | | | |

**Table S3:** Cheapest food items available by retail outlet type and country (urban, rural)

|  | |  | **Supermarket**  **urban, rural** | | **Independent Grocer**  **urban, rural** | | | | | **Convenience store**  **urban, rural** | | | | | **Informal vendor**  **urban, rural** | | | **Mobile vendor**  **urban, rural** | | | **Market**  **Urban, rural** | | |
| --- | --- | --- | --- | --- | --- | --- | --- | --- | --- | --- | --- | --- | --- | --- | --- | --- | --- | --- | --- | --- | --- | --- | --- |
|  |  | **UG** | **SA** | **SW** | **UG** | **SA** | | **SW** | | **UG** | **SA** | | | **SW** | **UG** | **SA** | **SW** | **UG** | **SA** | **SW** | **UG** | **SA** | **SW** |
| **Fruit:** cheapest per kg (intl.$) †  Type | U | 1,02 orange | 1,15 orange§ | 0,76 watermelon | - | - | | 2,08 bananas | | 7,81 apple | 1,65 satsuma | | | - | 1,82 guava¶ | 1,32 orange | 1,09 bananas | 1,86 tangerines |  |  | 1,40 papaya |  |  |
|  | R |  | 1,65  Orange | 1,75 satsuma | - | 0,82 apple | | 1,42 bananas | |  | - | | | 3,28 apple‡ | 0,93 orange | 1,65 banana |  | - | 1,65 bananas |  | 1,86 banana |  |  |
| **Veg:** cheapest per kg (intl.$)  Type | U | 0,47 cabbage | 0,66  carrot | 0,77 onion | - | - | | 1,10 onion | | - | 1,24 cabbage | | | - | 0,93 ntula | 1,24 carrot | 0,77 cabbage | - |  |  | 1,40 sweet potato |  |  |
|  | R |  | 0,66  cabbage | 0,76 cabbage | - | 0,58 onion | | 0,88 onion | |  | - | | | 1,85 onion | 0,37 eggplant | 2,48 onion |  | 0,93 cabbage | - |  | 0,28 eggplant |  |  |
| **Bread**  cheapest per package (intl.$)  brown/  white  *** SW-UG** # | U | 1,86 (500g) white | 0,99 (600g)  brown and white | 0,65 (250g) brown and white | - | 1,82 (700g) brown | | 0,66 (240g) white | | 2,33 (500g) white | 1,90 (700g) brown | | | - | - | 1,90 (700g) brown | - | - |  |  | 1,40 (100g), white |  |  |
|  | R |  | 0,99 (600g) brown | 0,87 (500g) brown | 2,04 (500g) white | 1,57 (600g) brown | | 0,36 (330g) brown and white | |  | 1,57 (600g) brown | | | 1,52 (260g) brown | 2,91 (500g) brown | - |  | - | - |  | - |  |  |
| **Milk**  cheapest per package (intl.$)  fat | U | 1,02 (200ml) full cream | 1,15 (1l) (medium fat = 1.6-3.3%) | 0,85 (1l) low fat 0,5% | 0,78 (200ml) not specified | 0,83 (1l) full cream | | 1,21 (1l) 1,5% , fat reduced fat | | 1,86 (250ml) full cream long life | 1,02 (250ml) full cream | | | - | - | 1,65 (1l) full cream | - | - |  |  | 1,49 (1l) full cream |  |  |
|  | R |  | 0,82 (250ml) full cream | 0,98 (1l) reduced fat (1,5%) | 0,47 (15g) full cream milk powder | 1,15 (250ml) full cream | | 1,21 (1l) 3,2% fat full cream | |  | 1,16 (250ml) full cream | | | 1,32 (1l) low fat 0,5% fat | - | - |  | - | - |  | - |  |  |
| **Yoghurt**  cheapest per package (intl.$)  plain/  sweetened  ***SW-SA** | U | 0,93 (125ml) sweetened | 1,15 (200ml) sweetened | 1,04 (1l) plain | - | 0,50 (100ml) sweetened | | 1,97 (1l) plain | | 1,86 (400ml) sweetened | 1,40 (200ml) sweetened | | | - | - | 0,50 (100ml) sweetened | - | - |  |  | 1,11 (500ml) sweetened |  |  |
|  | R |  | 0,99 (200ml) sweetened, low fat | 1,2 (1l) plain 0,5% fat | 1,86 (400ml) sweetened | - | | 1,64 (1l) plain | |  | 1,60 (200ml) sweetened | | | 1,63 (1l) plain | - | - |  | - | - |  | - |  |  |
| **Breakfast cereal**  cheapest per package (intl.$)  high fiber/source of/low fiber  Low /med/high sugar | U | 1,86 (250g) (not specified) | 1,07 (50g)  high fiber  medium sugar | 1,63 (750g) low sugar, high fiber | 2,13 (112g) low in sugar, source of fiber | 1,65 (220g) medium sugar, low fiber | | 1,86 (500g) medium sugar, low fiber | | 5,11 (1kg) not specified | 3,25 (500g) low sugar, high fiber | | | - | - | 2,81 (500g) low sugar, high fiber | - | - |  |  | - |  |  |
|  | R |  | 1,07 for (50g)  high fiber,  medium sugar | 1,09 (500g)  source of fiber, medium sugar | 3,26 (500g) low sugar, high fiber | 1,32 (1kg) medium sugar, low fiber | | 2,19 (375g) high in sugar, source of fiber | |  | 0,83 (150g) high sugar, source of fiber | | | 1,96 (750g) low sugar, high fiber | - | - |  | - | - |  | - |  |  |
| **Biscuits**  Cheapest per package (Intl.$)  ***SW-SA**  ***SW-UG** | U | 0,14 (not spec) | 0,17 (400g) | 1,08 (400g) | 0,69 (82g) | 0,08 (not spec) | | 0,55 (44g) | | 0,47 (43g) | 0,82 (150g) | | | 1,10 (40g) | - | 0,17 (30g) | - | - |  |  | - |  |  |
|  | R |  | 0,41 (50g) | 0,54 (200g) | 0,09 (11g) | 0,17 (40g) | | 0,55 (72g) | |  | 0,62 (50g) | | | 1,59 (200g) | - | 0,17 (not specified) |  | - | - |  | - |  |  |
| **Chips**  Cheapest per package (Intl.$)  ***SW-SA**  ***SW-UG** | U | 0,37 (24g) | 0,49  (20g) | 0,65 (40g) | 0,32 (24g) | 0,08 (22g) | | 1,10 (40g) | | 0,47 (24g) | 0,17 (20g) | | | 0,55 (40g) | - | 0,08 (20g) | - | - |  |  | - |  |  |
|  | R |  | 0,49  (30g) | 1,10 (200g) | - | 0,08 (16g) | | 0,55 (25g) | |  | 0,56 (14g) | | | 0,98 (35g) | - | 0,17 (20g) |  | - | - |  | - |  |  |
| **Chocolate bar**  Cheapest per package (Intl.$) | U | 1,31 (23g) | 0,41 (16g) | 0,43 (20g) | 0,27 (9g) | 0,33 (13g) | | 0,55 (25g) | | 0,93 (not specified) | 0,74 (not specified) | | | 1,10 (60g) | - | 0,08 (not specified) | - | - |  |  | - |  |  |
|  | R |  | 0,49  (16g) | 0,76 (60g) | 0,75 (18g) | 0,66 (17g) | | 0,77 (57g) | |  | 0,66 (20g) | | | 0,66 (24g) | - | - |  | - | - |  | - |  |  |
| **Fruit drink**  Cheapest per package (Intl.$)  Sweetened or 100% juice | U | 0,56 (300 ml) artificial sweeteners | 1,09 (250ml) | 0,66 (200ml) sugar sweetened | 0,58 (not spec) | 0,17 (150ml) artificial sweeteners | | 0,55 (200ml) sweetened | | 1,40 (250ml) sweetened | 0,83 (200ml) sweetened | | | 0,77 (200ml) sweetened | - | 1,65 (330ml) 100% fruit juice | - | - |  |  | 0,47 (300ml) |  |  |
|  | R |  | 0,91  (250ml)  100% juice | 0,87 (1l) 100% juice | 0,93 (300ml) sweetened | 0,82 (200ml) 100% juice | | 0,55 (200ml) sweetened | |  | 1,81 (250ml) 100% fruit juice | | | 0,88 (250ml) 100% fruit juice | - | - |  | - | - |  | - |  |  |
| **Soda, carbonated sweetened drink**  Cheapest per package (Intl.$) | U | 0,84 (320ml) | 0,79 (500ml) | 0,87 (330ml) | 1,16 (300ml) | 0,66 (300ml) | | 1,10 (330ml) | | 0,93 (330ml) | 1,24 (500ml) | | | 0,77 (330ml) | - | 1,49 (440ml) | - | - |  |  | 0,93 (330ml) |  |  |
|  | R |  | 0,82  (300ml) | 0,76 (330ml) | 0,93 (320ml) | 0,82 (330ml) | | 0,77 (331ml) | |  | 1,49 (330ml) | | | 1,49 (330ml) | 0,93 (300ml) | - |  | - | - |  | - |  |  |
| **Energy drink**  Cheapest per package (Intl.$)  ***SW-UG** | U | 1,67 (320ml) | 1,32 (500ml) | 0,43 (250ml) | 2,09 (250ml) | 1,65 (500ml) | | 1,10 (250ml) | | 1,86 (320ml) | 0,64 (440ml) | | | 1,10 (250ml) | - | 1,65 (500ml) | - | - |  |  | 1,86 (320ml) |  |  |
|  | R |  | 1,15  (440ml) | 0,76 (250ml) | 0,18 (320ml) | 1,15 (400ml) | | 0,55 (250ml) | |  | 1,82 (500ml) | | | 2,41 (250ml) | 1,86 (320ml) | - |  | - | - |  | - |  |  |
|  |  |  |  |  | | |  | |  | | |  |  | | | | | | | | | | |

†Conversion rates: SA: /6.060; SW: /9.125; UG: /1098 (+2.1%)

‡ 1,85 Intl.$ (16,90 SEK) lemon, replaced since lemons are used as a condiment.

§ 0, 66 Intl.$ (3.99 ZAR) lemon, replaced since lemons are used as a condiment.

¶ 0,47 Intl.$ (500 UGX) lemon, replaced since lemons are used as a condiment.

# *p* values based on Independent samples Kruskal-Wallis test to compare prices of food items across countries

* ≤ 0,05 with significance level. *SW-UG/SW-SA indicates a significant relationship between countries

- signifies unavailability of that item

|  | A black box indicates that the retail outlet is not present. |
| --- | --- |

| \| **Table S4:** Packaged food product labelling - Uganda \| \| \| \| \| \| \| \| \| \| \| --- \| --- \| --- \| --- \| --- \| --- \| --- \| --- \| --- \| --- \| \|  \| \|  \| **Back-of-pack label** \| \| \| \| **Front-of-pack label** \| \| \| \|  \| \| **Products with a package** \| **Nutrition info in required language**† \| \| **Ingredients list** \| **Nutrition facts** \| **Consumer guidance info** \| **Nutrition Claim** \| **Health Claim** \| \| **Urban** \|  \| \| \| \| \| \| \| \| \| \| **Supermarket** \| \| n=22 \| 21 (95.5%) \| \| 17 (77.3%) \| 15 (68.2%) \| 3 (13.6%) \| 3 (13.6%) \| 9 (40.9%) \| \| **Independent Grocer** \| \| n=14 \| 12 (85.7%) \| \| 10 (71.4%) \| 11 (78.6%) \| 2 (14.3%) \| 3 (21.4%) \| 2 (14.3%) \| \| **Convenience store** \| \| n=17 \| 17 (100%) \| \| 14 (82.3%) \| 14 (82.3%) \| 1 (5.9%) \| 3 (17.6%) \| 5 (29.4%) \| \| **Informal vendor** \| \| n=0 \| 0 \| \| 0 \| 0 \| 0 \| 0 \| 0 \| \| **Mobile vendor** \| \| n=0 \| 0 \| \| 0 \| 0 \| 0 \| 0 \| 0 \| \| **Market** \| \| n=4 \| 3 (75%) \| \| 3 (75%) \| 3 (75%) \| 0 (0%) \| 0 (0%) \| 1 (25%) \| \| **Urban total** \| \| n=57 \| 53 (92.9%) \| \| 44 (77.2%) \| 43 (75.4%) \| 6 (10.5%) \| 9 (15.8%) \| 17 (29.8%) \| \| **Rural** \|  \| \| \| \| \| \| \| \| \| \| **Supermarket** \| \|  \| \|  \| \| \| \| \| \| \| **Independent Grocer** \| \| n=17 \| 17 (100%) \| \| 15 (88.2%) \| 12 (70.6% \| 2 (11.8%) \| 4 (23.5%) \| 5 (29.4%) \| \| **Convenience store** \| \|  \| \|  \| \| \| \| \| \| \| **Informal vendor** \| \| n=8 \| 8 (100%) \| \| 8 (100%) \| 5 (62.5%) \| 0 (0%) \| 1 (12.5%) \| 1 (12.5%) \| \| **Mobile vendor** \| \| n=0 \| 0 \| \| 0 \| 0 \| 0 \| 0 \| 0 \| \| **Market** \| \| n=0 \| 0 \| \| 0 \| 0 \| 0 \| 0 \| 0 \| \| **Rural total** \| \| n=25 \| 25 (100%) \| \| 23 (92.0%) \| 17 (68%) \| 2 (8%) \| 5 (20.0%) \| 6 (24.0%) \| \| †The categories are not mutually exclusive. \| \| \| \| \| \| \| \| \| \|   **Table S5:** Packaged food product labelling - South Africa | | | | | | | | |
| --- | --- | --- | --- | --- | --- | --- | --- | --- | --- | --- | --- | --- | --- | --- | --- | --- | --- | --- | --- | --- | --- | --- | --- | --- | --- | --- | --- | --- | --- | --- | --- | --- | --- | --- | --- | --- | --- | --- | --- | --- | --- | --- | --- | --- | --- | --- | --- | --- | --- | --- | --- | --- | --- | --- | --- | --- | --- | --- | --- | --- | --- | --- | --- | --- | --- | --- | --- | --- | --- | --- | --- | --- | --- | --- | --- | --- | --- | --- | --- | --- | --- | --- | --- | --- | --- | --- | --- | --- | --- | --- | --- | --- | --- | --- | --- | --- | --- | --- | --- | --- | --- | --- | --- | --- | --- | --- | --- | --- | --- | --- | --- | --- | --- | --- | --- | --- | --- | --- | --- | --- | --- | --- | --- | --- | --- | --- | --- | --- | --- | --- | --- | --- | --- | --- | --- | --- | --- | --- | --- | --- | --- | --- | --- | --- | --- | --- | --- | --- | --- | --- | --- | --- | --- | --- | --- | --- | --- | --- | --- | --- | --- | --- | --- | --- | --- | --- | --- | --- | --- | --- | --- | --- | --- | --- | --- | --- | --- | --- | --- | --- | --- | --- | --- | --- | --- | --- | --- | --- | --- | --- | --- | --- | --- | --- | --- | --- | --- | --- | --- | --- | --- | --- | --- | --- | --- | --- | --- | --- |
|  |  | **Back-of-pack label** | | | | **Front-of-pack label** | | |
|  | **Products with a package** | **Nutrition info in required language**† | | **Ingredients list** | **Nutrition facts** | **Consumer guidance info** | **Nutrition claim** | **Health claim** |
| **Urban** | | | | | | | | |
| **Supermarket** | n=22 | 20 (90.9%) | | 17 (77.3%) | 17 (77.3%) | 2 (9%) | 10 (45.5%) | 7 (31.8%) |
| **Independent Grocer** | n=21 | 18 (85.7%) | | 16 (76.2%) | 16 (76.2%) | 4 (19%) | 7 (33.3%) | 3 (14.3%) |
| **Convenience store** | n=22 | 21 (95.5%) | | 19 (86.4%) | 19 (86.4%) | 7 (31.8%) | 10 (45.5%) | 5 (22.7%) |
| **Informal vendor** | n=11 | 10 (90.9%) | | 9 (81.8%) | 9 (81.8%) | 5 (45.5%) | 5 (45.5%) | 3 (27.3%) |
| **Mobile vendor** |  | |  | | | | | |
| **Market** |  | |  | | | | | |
| **Urban total** | n=76 | 69 (90.8%) | | 61 (80.3%) | 61 (80.3%) | 18 (23.7%) | 32 (42.1%) | 18 (23.7%) |
| **Rural** | | | | | | | | |
| **Supermarket** | n=22 | 20 (90.9%) | | 19 (86.4%) | 18 (81.8%) | 3 (13.6%) | 9 (40.9%) | 3 (13.6%) |
| **Independent Grocer** | n=18 | 18 (100%) | | 15 (83.3%) | 15 (83.3%) | 3 (16.7%) | 6 (33.3%) | 5 (27.8%) |
| **Convenience store** | n=20 | 20 (100%) | | 17 (85%) | 20 (100%) | 6 (30%) | 7 (35%) | 5 (25%) |
| **Informal vendor** | n=3 | 2 (66.7%) | | 2 (66.7%) | 2 (66.7%) | 0 (0%) | 1 (33.3%) | 0 (0%) |
| **Mobile vendor** | n=0 | 0 | | 0 | 0 | 0 | 0 | 0 |
| **Market** |  | |  | | | | | |
| **Rural total** | n=63 | 60 (95.2%) | | 53 (84.1%) | 55 (87.3%) | 12 (19.0%) | 23 (36.5%) | 13 (20.6%) |
| †The categories are not mutually exclusive. | | | | | | | | |

| **Table S6:** Packaged food product labelling - Sweden | | | | | | | | | |
| --- | --- | --- | --- | --- | --- | --- | --- | --- | --- |
|  | |  | **Back-of-pack label** | | | | **Front-of-pack label** | | |
|  | | **Products with a package** | **Nutrition info in required language**† | | **Ingredients list** | **Nutrition facts** | **Consumer guidance info** | **Nutrition claim** | **Health claim** |
| **Urban** |  | | | | | | | | |
| **Supermarket** | | n=22 | 21 (95.5%) | | 19 (86.4%) | 20 (90.9%) | 9 (40.9%) | 6 (27.3%) | 1 (4.5%) |
| **Independent Grocer** | | n=21 | 20 (95.2%) | | 19 (90.5%) | 20 (95.2%) | 12 (57.1%) | 3 (14.3%) | 3 (14.3%) |
| **Convenience store** | | n=12 | 11 (91.7%) | | 11 (91.7%) | 11 (91.7%) | 5 (41.7%) | 1 (8.3%) | 1 (8.3%) |
| **Informal vendor** | | n=0 | 0 | | 0 | 0 | 0 | 0 | 0 |
| **Mobile vendor** | |  | |  | | | | | |
| **Market** | |  | |  | | | | | |
| **Urban total** | | n=55 | 52 (94.5%) | | 49 (89.1%) | 51 (92.7%) | 26 (47.2%) | 10 (18.2%) | 5 (9.1%) |
| **Rural** |  | | | | | | | | |
| **Supermarket** | | n=22 | 22 (100%) | | 20 (90.9%) | 22 (100%) | 12 (54.5%) | 2 (9.1%) | 1 (4.5%) |
| **Independent Grocer** | | n=19 | 19 (100%) | | 18 (94.7%) | 18 (94.7%) | 5 (26.3%) | 1 (5.3%) | 0 (0%) |
| **Convenience store** | | n=21 | 20 (95.2%) | | 18 (85.7%) | 20 (95.2%) | 11 (52.4%) | 6 (28.6%) | 2 (9.5%) |
| **Informal vendor** | |  | |  | | | | | |
| **Mobile vendor** | |  | |  | | | | | |
| **Market** | |  | |  | | | | | |
| Rural total | | n=62 | 61 (98.4%) | | 56 (90.3%) | 60 (96.7%) | 28 (45.1%) | 9 (14.5%) | 3 (4.8%) |
| †The categories are not mutually exclusive. | | | | | | | | | |
